# Circulating CD3^+^CD4^+^CD161^+^ Cells Are Associated with Early Complications after Autologous Stem Cell Transplantation in Multiple Myeloma

**DOI:** 10.1155/2018/5097325

**Published:** 2018-01-01

**Authors:** Sung-Eun Lee, Ji-Young Lim, Da-Bin Ryu, Tae Woo Kim, Young-Woo Jeon, Jae-Ho Yoon, Byung-Sik Cho, Ki-Seong Eom, Yoo-Jin Kim, Hee-Je Kim, Seok Lee, Seok-Goo Cho, Dong-Wook Kim, Jong Wook Lee, Woo-Sung Min, Chang-Ki Min

**Affiliations:** ^1^Department of Hematology, Seoul St. Mary's Hospital, College of Medicine, The Catholic University of Korea, Seoul, Republic of Korea; ^2^Catholic Leukemia Research Institute, The Catholic University of Korea, Seoul, Republic of Korea

## Abstract

The aim of this study was to explore if measurement of pretransplant circulating CD161-expressing cells, in addition to clinical risk factors, could predict mucositis and infections in patients with multiple myeloma (MM) undergoing autologous stem cell transplantation (ASCT). To determine if CD161-expressing cells are likely to predict early complications, namely, mucositis (≥grade 3), infections, and cytomegalovirus (CMV) reactivation, we prospectively examined CD161-expressing cells (CD3^+^CD4^+^CD161^+^ and CD3^+^CD8^+^CD161^+^) in peripheral blood samples from 108 patients with MM undergoing ASCT. After adjusting for factors identified by univariate analysis that predicted mucositis (≥grade 3), infection before engraftment, and CMV reactivation, multivariate analyses revealed that the low proportion of CD3^+^CD4^+^CD161^+^ cells in peripheral blood was an independent predictor of mucositis (≥grade 3) (*P* = 0.020), infections before engraftment (*P* = 0.014), and CMV reactivation (*P* = 0.010). In addition, we found that female sex and decreased glomerular filtration rate were independent factors for predicting mucositis. Female sex and severe pulmonary comorbidity were independent factors for predicting infection before engraftment. We found that the proportion of circulating CD3^+^CD4^+^CD161^+^ cells is useful for predicting the occurrence of early complications, including mucositis and infections, after ASCT in patients with MM.

## 1. Introduction

High-dose therapy followed by autologous stem cell transplantation (ASCT) is the standard of care for transplant-eligible patients with multiple myeloma (MM) [1–4]. Although dose intensification of melphalan yields therapeutic effects, this approach also results in treatment-related toxicities such as mucositis and infections, which are the most important causes of treatment-related complications [5, 6]. Infections during the profound neutropenia in the preengraftment phase of transplantation can progress rapidly, leading to other life-threatening complications. Moreover, patients with MM are at high risk of both bacterial and viral infections due to patient- and disease-related factors as well as treatment-related factors [7]. Therefore, it is critical to recognize neutropenic fever early and to promptly initiate empiric systemic antibacterial therapy to avoid mortality.

The innate immune system is a first line of defense against pathogens and sterile injury [8]. Innate immune cells including monocytes, macrophages, neutrophils, NK cells, NKT cells, and *γδ* cells recognize broadly expressed molecules derived from pathogens or apoptotic cells, and their activation plays an important role in priming adaptive immune responses. Considering that before the initial engraftment a patient's remaining innate immune system may play defensive roles against infections, we previously evaluated the influence of a circulating innate T cell subtype, CD161-expressing T cells, on early outcomes after allogeneic SCT. In particular, circulating CD3^+^CD4^+^CD161^+^ cell proportion was a risk factor for the occurrence of clinically or microbiologically documented infections before engraftment [9].

Although the influence of CD161-expressing T cells on early complications after allogeneic SCT has been examined [9], their role in early complications after ASCT in patients with MM, who have different medical conditions and immune system characteristics, are uncertain. In this study, we explored the ability of circulating CD161-expressing cells (as detected prior to ASCT) to predict mucositis and infections in patients with MM undergoing ASCT. In addition to the immune parameters, we also investigated clinical risk factors that could predict patient outcomes.

## 2. Materials and Methods

### 2.1. Patient Selection

A total of 108 consecutive patients with MM who underwent ASCT as part of front-line treatment at our institution between January 2012 and December 2015 were included in this analysis. To identify novel immune cell biomarkers predictive of early complications, we prospectively obtained peripheral blood samples before they underwent conditioning chemotherapy. In addition, various clinical and laboratory data that we hypothesized may contribute to the early development of complications following ASCT collected before the conditioning regimen was initiated. All patients received a pulmonary function test, echocardiography, liver Doppler ultrasound, and infection assessment prior to ASCT. The Institutional Review Board of the Catholic University of Korea approved the research protocol for all data analysis. This study was also conducted in accordance with the Declaration of Helsinki.

### 2.2. Transplant Procedures

ASCT was performed after achieving a response greater than a partial response (PR). Some patients without progressive MM who were resistant to various agents (bortezomib and thalidomide) underwent ASCT. General ASCT procedures were performed as described previously [10, 11]. Briefly, all patients were mobilized with cyclophosphamide (3 g/m^2^ total) for 2 days and then treated subcutaneously once daily with G-CSF (lenograstim, JW Pharmaceutical, Seoul, Korea) at 10 *μ*g/kg/day. Conditioning consisted of melphalan (100 mg/m^2^) for 2 days except in patients with serum creatinine above 2.0 mg/dl or on hemodialysis, for whom 70 mg/m^2^ for 2 days was used. G-CSF (5 *μ*g/kg/day) was given subcutaneously to all patients from one day after transplantation until the absolute neutrophil count (ANC) reached >3.0 × 10^9^/L. All patients received prophylactic ciprofloxacin and an antifungal agent (micafungin) starting 4 days before transplantation and continued to receive these drugs until the ANC reached 1.5 × 10^9^/L.

### 2.3. Isolation of Mononuclear Cells and Flow Cytometric Analysis

Blood samples for cell population analysis were collected one day before conditioning chemotherapy. Peripheral blood mononuclear cells (PBMCs) were isolated from whole blood samples (10 mL) collected in EDTA-coated tubes by centrifugation in Ficoll-Paque Plus. PBMCs were processed immediately for analysis. Flow cytometry was used to evaluate the percentages of CD3^+^, CD4^+^CD161^+^, and CD8^+^CD161^+^ T cells; natural killer (NK) cells (CD16^+^CD56^+^); and myeloid-derived suppressor cells (MDSCs) [Lin^−^HLA-DR^−^CD11b^+^CD33^+^ (granulocytic) and HLA-DR^−^CD14^+^ (monocytic)]. Anti-CD3-allophycocyanin (APC), anti-CD4-fluorescein isothiocyanate (FITC), anti-CD8-phycoerythrin (PE), anti-CD161-PerCP-Cy5.5, anti-CD16-FITC, anti-CD56-PE, and anti-CD14-APC monoclonal antibodies (mAbs) were purchased from eBioscience (San Diego, CA, USA). Anti-Lineage cocktail 1 (Lin 1)-FITC, anti-HLA-DR-PerCP, rat anti-mouse CD11b-APC-Cy™7, and mouse anti-human CD33-V450 (BD BioSciences) mAbs were purchased from BD Biosciences (San Jose, CA). CD4^+^CD161^+^ and CD8^+^CD161^+^ T cells were gated on CD3^+^ cells and are expressed as percentages of lymphocytes. The frequencies of HLA-DR^−^Lin^−^CD11b^+^CD33^+^ and HLA-DR^−^CD14^+^ MDSCs are expressed as percentages of total PBMCs. Flow cytometry was performed using a FACS LSR Fortessa (BD Biosciences).

### 2.4. Definitions

Toxicity was classified using the National Cancer Institute Common Toxicity Criteria grading scheme. Infections included microbially and clinically defined infections as proposed by the Immunocompromised Host Society [12, 13], and in this study, fevers of unknown origin were excluded. For surveillance of cytomegalovirus (CMV) reactivation, quantitative real-time PCR (qPCR) for CMV DNA after neutrophil engraftment was tested using a LightCycler® 2.0 instrument (Roche Diagnostics, Mannheim, Germany). CMV reactivation was diagnosed when a patient previously found to be CMV seropositive exhibited >500 copies/mL by qPCR. Early clinical complications assessed were mucositis (≥grade 3), infection before engraftment, and CMV reactivation after engraftment. Severity of comorbidities was classified using definitions from the hematopoietic cell transplantation-specific comorbidity index [14].

### 2.5. Statistical Analysis

Potential risk factors for the occurrence of early complications in patients with MM undergoing ASCT were assessed using logistic regression analysis. Optimal cutoffs for continuous variables were identified using receiver operating characteristic (ROC) curve analysis. To investigate whether the identified markers were independent predictors, covariates with a *P* value less than 0.1 in the univariate analyses were added to the multivariate analysis model. Student's *t*-test was used to compare continuous variables.

## 3. Results

### 3.1. Patient Characteristics and Early Complications

A total of 108 patients with MM were analyzed, of whom 64 (59%) were male and 44 (41%) were female. The median age was 56 years (range, 32–67 years) and the median disease duration before ASCT was 6.8 months (range, 2.9–21.6 months). Demographic information for all patients is listed in Table 1.

Among the 108 patients, 54 patients (50%) developed mucositis, including 25 with grade 2 and 29 with ≥grade 3. Analysis of risk factors was performed for mucositis (≥grade 3), because mucositis (≥grade 3) had a substantial effect on infection risk. The incidence of infection before engraftment was 52%. Twenty-nine patients (27%) developed CMV reactivation, nine of whom received ganciclovir therapy. One patient died due to severe sepsis within 100 days.

### 3.2. Analysis of CD161^+^ T Cell Count prior to ASCT

**Figure 1:**
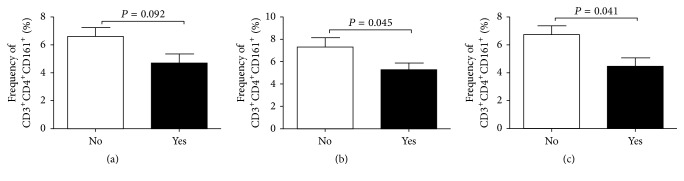
Proportions of pretransplant CD3^+^CD4^+^CD161^+^ cells according to (a) the occurrence of mucositis (≥grade 3), (b) infection before engraftment, and (c) CMV reactivation.

The median MNC count was 1.2 × 10^6^ cells/mL (range, 0.2–3.4 × 10^6^ cells/mL), and the mean (±standard error) proportions of CD3^+^CD4^+^CD161^+^ T cells and CD3^+^CD8^+^CD161^+^ T cells were 6.10 ± 0.50% and 8.67 ± 0.91%, respectively. The proportions of CD3^+^CD4^+^CD161^+^ and CD3^+^CD8^+^CD161^+^ T cells were compared according to the occurrence of mucositis (≥grade 3), infection before engraftment, and CMV reactivation (Figure 1). The proportion of CD3^+^CD4^+^CD161^+^ T cells was lower in patients who developed infection before engraftment (5.28 ± 0.60% versus 7.32 ± 0.83%, *P* = 0.045) and CMV reactivation (4.46 ± 0.61% versus 6.74 ± 0.63%, *P* = 0.041). Patients with mucositis ≥ grade 3 tended to have a lower proportion of CD3^+^CD4^+^CD161^+^ T cells (4.70 ± 0.65% versus 6.61 ± 0.63%, *P* = 0.092). In contrast, the proportion of CD3^+^CD8^+^CD161^+^ T cells was not associated with early complications.

In addition, although we compared the proportions of NK cells and MDSCs according to the occurrence of mucositis (≥grade 3), infection before engraftment, and CMV reactivation in 59 patients with available data, there were no significant associations with early complications (Supplementary Table 1).

### 3.3. Risk Factors for Mucositis (≥Grade 3)

The results of univariate analysis for potential risk factors predicting the development of mucositis (≥grade 3) following ASCT are listed in Table 2. To explicitly assess renal comorbid condition in patients with MM, both serum creatinine and glomerular filtration rate (GFR), which was calculated using the modification of diet in renal disease (MDRD), were included as continuous variables. Decreased proportion of CD3^+^CD4^+^CD161^+^ T cells was associated with the occurrence of mucositis (≥grade 3). Female sex, decreased body mass index (BMI), and decreased GFR were also factors that predicted the occurrence of mucositis (≥grade 3).

**Figure 2:**
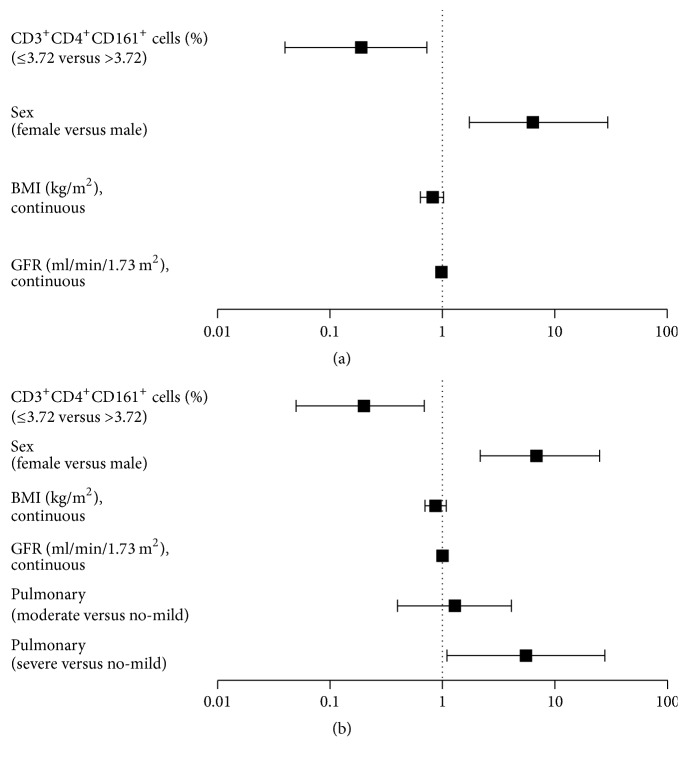
Multivariate analyses including clinical risk factors showing the predictive role of the proportion of pretransplant CD3^+^CD4^+^CD161^+^ cells for (a) the occurrence of mucositis (≥grade 3) and (b) infection before engraftment.

In multivariate analysis (Table 3), stratification of patients into low and high groups based on the proportion of CD3^+^CD4^+^CD161^+^ T cells by ROC curve analysis revealed a significant protective role for the proportion of pretransplant CD3^+^CD4^+^CD161^+^ cells against the occurrence of mucositis (≥grade 3) (cutoff of 3.72%, relative risk (RR) of 0.19, *P* = 0.020). In addition, female sex (*P* = 0.009) and decreased GFR (*P* = 0.020) were independent risk factors. Patients with decreased BMI tended to have a higher mucositis rate (*P* = 0.094) (Figure 2(a)).

### 3.4. Risk Factors for Infection before Engraftment and CMV Reactivation

Female sex, decreased BMI, decreased GFR, and severe pulmonary comorbidity predicted the occurrence of infection before engraftment. In addition, decreased BMI predicted the occurrence of CMV reactivation (Table 2). These clinical factors were included in multivariate analysis (Table 3). The low proportion of CD3^+^CD4^+^CD161^+^ T cells was significantly associated with a higher risk of infection before engraftment (cutoff of 3.72%, RR of 0.20, and *P* = 0.014) and CMV reactivation (cutoff of 3.72%, RR of 0.25, and *P* = 0.010). In addition, female sex and severe pulmonary comorbidity were associated with the occurrence of infection before engraftment (*P* = 0.020 and *P* = 0.038, resp.) (Figure 2(b)).

## 4. Discussion

Myeloablative high-dose chemotherapy followed by ASCT prolongs survival for patients with MM [1, 2, 15, 16]. Although the value of high-dose chemotherapy has been debated for years because of a meta-analysis that showed no clear overall survival benefit [17], ASCT as a first-line treatment is still the standard of care, and novel agents have markedly improved response depth before and after ASCT [18–21]. However, because of the toxic effects of ASCT and the advanced age of many patients with MM, accurate assessment of eligibility is crucial [22]. Moreover, in addition to the toxicities associated with ASCT, patients with MM are susceptible to infection [23] due to dysfunctions in humoral immunity and various immune system including T cells [24–26], dendritic cells [27], and natural killer cells [28]. In addition, the preengraftment period is particularly important in ASCT because the rapid reconstitution of humoral and cellular immunity results in a reduced risk of opportunistic infection by 3 months after ASCT. Therefore, we focused on patient and transplant characteristics that we thought might be important for predicting early complications in patients with MM undergoing ASCT. In particular, we explored the influence of circulating CD161-expressing T cell populations as measured prior to ASCT on early complications based on the reason that, before the initial engraftment, the patient's remaining immune system may defend against mucositis and subsequent infections, particularly during profound neutropenia.

CD161 is a type II transmembrane glycoprotein with characteristics of the C-type lectin superfamily. This receptor is expressed on NK cells, 25% of all adult peripheral T cells, more than 90% of all peripheral blood monocytes, and on* in vitro*-derived dendritic cells [29]. We previously evaluated the association of CD161-expressing T cells with the development of acute graft-versus-host disease after allogeneic SCT and found that a low proportion of CD8^+^CD161^+^ cells and a high ratio of CD4^+^CD161^+^ cells to CD8^+^CD161^+^ cells from peripheral blood at engraftment were associated with the occurrence of acute graft-versus-host disease, with evidence of higher expression of the Th17 transcription factor ROR*γ*T in CD4^+^CD161^+^ T cells rather than CD8^+^CD161^+^ T cells [30]. Furthermore, we reported that in 282 patients receiving allogeneic SCT pretransplant circulating CD3^+^CD4^+^CD161^+^ cell proportion was associated with the occurrence of neutropenic infections, suggesting the importance of CD3^+^CD4^+^CD161^+^ cells in a rapid immune response to microorganisms before establishment of the posttransplant immune system [9]. In the present study, we showed that pretransplant proportion of CD3^+^CD4^+^CD161^+^ T cells in patients with MM undergoing ASCT can predict the occurrence of early complications, suggesting that CD161-expressing T cells play an important role in the inflammatory milieu.

In this study, we sought to identify predictors of mucositis in a homogeneous group of patients with MM undergoing ASCT following high-dose melphalan treatment, because mucositis risk is believed to vary according to the conditioning regimen intensity, SCT type (autologous versus allogeneic), and patient-related factors. Regarding SCT-associated mucositis, previous studies have reported that significant oral mucositis occurs in about 75% of all patients undergoing SCT [31–33] according to the type of chemotherapeutic agent, with high-dose melphalan known to cause severe mucositis [32]. Importantly, severe mucositis not only extends hospitalization and increases cost, but also may be associated with increased mortality. Rapoport et al. showed that severe mucositis was correlated with increased incidences of bacteremia and mortality [34]. In their study, 60% of all patients with a peak mucositis score of 18 or higher according to the Oral Assessment Guide (OAG) [35] developed bacteremia, compared with 30% of all patients with peak scores lower than 18 (*P* = 0.001). Additionally, an association between infection and severe mucositis was supported by a study that reported that streptococcal infections were three times more prevalent in patients with severe ulcerative mucositis than patients with mild symptoms [36]. In this study, we also found a substantial effect of severe mucositis on infection risk during the preengraftment period (data not shown) and identified a significant protective role for pretransplant CD3^+^CD4^+^CD161^+^ T cells against the occurrence of mucositis (≥grade 3), in addition to clinical factors including female sex and decreased GFR. Using biomarkers as predictors of early complications in ASCT, we can assess transplant eligibility and identify those patients most likely to benefit from dose modification of conditioning chemotherapy and additional prophylactic treatments. Although the clinical predictors found in our study can also help establish treatment strategies to reduce excessive transplant toxicities, obviously we have several limitations of the small size of patient population and missed risk factors.

CMV reactivation has been demonstrated to be relatively common after ASCT for MM (48.5%), especially when tandem transplantation is performed using combination chemotherapy with high-dose melphalan [37]. We observed CMV reactivation in approximately 27% of all patients and found that the proportion of pretransplant CD3^+^CD4^+^CD161^+^ T cells was lower in patients with CMV reactivation than those without CMV reactivation. Kang et al. analyzed CD161 expression and cytokine secretion in CD4^+^ T cells in blood and liver samples from patients infected with hepatitis C. They observed marked enrichment of CD161^+^CD4^+^ T cells in the liver during chronic hepatitis and a population of CD4^+^ T cells cosecreting IL-22 and IFN-*γ* [38]. Therefore, further studies are required to characterize the phenotypes of these cells and to assess their immunological functions in viral inflammation.

## 5. Conclusions

We identified that circulating CD3^+^CD4^+^CD161^+^ T cells, in addition to clinical risk factors, were associated with the occurrence of early complications in the setting of ASCT in patients with MM. Pretransplant predictive immune markers that can predict the occurrence of early complications will help assess transplant eligibility and identify those patients who require dose modification of conditioning chemotherapy and intensified prophylactic strategies.

## Figures and Tables

**Table 1:** Patient characteristics.

Characteristic	Total (*N* = 108) (%)
Age, years, median (range)	56 (32–67)
Sex (M/F)	64 (59)/44 (41)
BMI (kg/m^2^), median (range)	24.6 (17.6–33.0)
Serum M-protein	
IgG, kappa	28 (26)
IgG, lambda	31 (29)
IgA, kappa	5 (5)
IgA, lambda	9 (8)
Light chain, kappa	11 (10)
Light chain, lambda	17 (16)
Other	7 (6)
Durie-Salmon stage	
I-II	14 (13)
IIIA/B	73 (68)/21 (19)
Cytogenetics^*∗*^	
Standard risk/high risk/NA	38 (35)/46 (43)/24 (22)
Myeloma bone disease on plain radiographs, yes/no	64 (59)/44 (41)
Creatinine at diagnosis, mg/dL, median (range)	1.02 (0.55–11.61)
*β*2-microglobulin at diagnosis, mg/mL, median (range)	3.46 (0.2–44.0)
Duration from diagnosis to ASCT, months, median (range)	6.8 (2.9–21.6)
*At the time of ASCT*	
Creatinine, mg/dL, median (range)	0.70 (0.34–10.28)
GFR^†^, ml/min/1.73 m^2^, median (range)	104.62 (5.11–208.22)
Diabetes	13 (12)
Arrhythmia/cardiac/heart valve disease	2 (2)/5 (5)/0 (0)
Pulmonary	
Mild/moderate/severe	17 (16)/45 (42)/25 (23)
Hepatic	
Mild/moderate-severe	15 (14)/0 (0)
HCT-CI	
0–4/≥4	63 (58)/45 (42)

ASCT, autologous stem cell transplantation; BMI, body mass index; GFR, glomerular filtration rate; HCT-CI, hematopoietic cell transplantation comorbidity index; NA, not available. ^*∗*^High-risk cytogenetics was defined as hypodiploidy or deletion of chr13 on conventional cytogenetics or presence of t(4;14), t(14;16), and -17p on fluorescent in situ hybridization and/or conventional cytogenetics. All other cytogenetic abnormalities were considered standard risk. ^†^Modification of diet in renal disease (MDRD) GFR was used.

**Table 2:** Univariate analyses of risk factors for the occurrence of mucositis, infection before engraftment, and CMV reactivation.

Variable	Mucositis (≥grade 3)		Infection before engraftment (CDI + MDI)		CMV reactivation	
	RR (95% CI)	*P*	RR (95% CI)	*P*	RR (95% CI)	*P*
Age (years), continuous	1.03 (0.98–1.09)	0.272	1.00 (0.95–1.04)	0.846	1.04 (0.99–1.11)	0.149
Sex (female versus male)	2.73 (1.14–6.53)	0.024	2.25 (1.02–4.95)	0.044	0.57 (0.23–1.40)	0.216
BMI (kg/m^2^), continuous	0.84 (0.72–0.99)	0.037	0.86 (0.75–0.99)	0.039	0.87 (0.75–1.02)	0.088
Diabetes (yes versus no)	0.46 (0.10–2.20)	0.330	1.10 (0.34–3.50)	0.878	1.85 (0.55–6.20)	0.319
Cr (mg/dl), continuous	1.10 (0.77–1.56)	0.617	1.59 (0.81–3.10)	0.178	1.15 (0.81–1.65)	0.433
GFR^*∗*^ (ml/min/1.73 m^2^), continuous	0.98 (0.97–0.99)	0.006	0.99 (0.98–1.00)	0.096	1.00 (0.99–1.01)	0.450
Pulmonary						
None-mild	1		1		1	
Moderate	2.21 (0.79–6.19)	0.130	0.89 (0.37–2.12)	0.790	1.52 (0.55–4.19)	0.415
Severe	1.72 (0.52–5.71)	0.374	2.86 (0.97–8.42)	0.057	1.77 (0.56–5.55)	0.332
Hepatic						
None	1		1		1	
Mild	2.03 (0.65–6.32)	0.222	0.41 (0.13–1.30)	0.130	0.64 (0.17–2.47)	0.521
Moderate-severe	-	-	-	-	-	-
HCT-CI						
0–4	1		1		1	
≥4	0.89 (0.29–2.71)	0.836	3.44 (1.15–10.28)	0.027	0.89 (0.29–2.71)	0.836
Proportion of CD3^+^CD4^+^CD161^+^ cells (%), continuous	0.88 (0.76–1.02)	0.098	0.90 (0.80–1.00)	0.055	0.86 (0.73–1.00)	0.047
Proportion of CD3^+^CD8^+^CD161^+^ cells (%), continuous	0.99 (0.93–1.06)	0.781	0.97 (0.92–1.03)	0.352	0.94 (0.88–1.02)	0.129

BMI, body mass index; CDI, clinically defined infection; CI, confidence interval; CMV, cytomegalovirus; GFR, glomerular filtration rate; HCT-CI, hematopoietic cell transplantation comorbidity index; MDI, microbially defined infection; RR, relative risk. ^*∗*^Modification of diet in renal disease (MDRD) GFR was used.

**Table 3:** Multivariate analyses of risk factors for the occurrence of mucositis, infection before engraftment, and CMV reactivation.

Variable	Mucositis (≥grade 3)		Infection before engraftment (CDI + MDI)		CMV reactivation	
	RR (95% CI)	*P*	RR (95% CI)	*P*	RR (95% CI)	*P*
CD3^+^CD4^+^CD161^+^ cells (%) (≤3.72 versus >3.72)	0.19 (0.04–0.73)	0.020	0.20 (0.06–0.73)	0.014	0.25 (0.09–0.72)	0.010
Sex (female versus male)	6.39 (1.74–29.71)	0.009	6.87 (2.05–23.04)	0.020	-	-
BMI (kg/m^2^), continuous	0.82 (0.64–1.03)	0.094	0.87 (0.71–1.08)	0.213	0.95 (0.78–1.16)	0.595
GFR^*∗*^ (ml/min/1.73 m^2^), continuous	0.98 (0.97–1.00)	0.020	1.00 (0.98–1.01)	0.502	-	-
Pulmonary						
None/mild	-	-	1		-	-
Moderate	-	-	1.29 (0.40–4.12)	0.671	-	-
Severe	-	-	5.54 (1.10–27.85)	0.038	-	-

BMI, body mass index; CDI, clinically defined infection; CI, confidence interval; CMV, cytomegalovirus; GFR, glomerular filtration rate; MDI, microbially defined infection; RR, relative risk. ^*∗*^Modification of diet in renal disease (MDRD) GFR was used.

## Supplementary Materials

Supplementary Materials Supplementary Table  1: pretransplant cell populations according to the occurrence of mucositis, infection, and CMV reactivation.
