# Supplementary material for: Circulating CD3+CD4+CD161+ Cells Are Associated with Early Complications after Autologous Stem Cell Transplantation in Multiple Myeloma
Source: Biomed Res Int. 2018 Jan 1;2018:5097325. doi: 10.1155/2018/5097325 (PMC5817817; doi:10.1155/2018/5097325)
Supplement: Supplementary Materials — Supplementary Table 1: pretransplant cell populations according to the occurrence of mucositis, infection, and CMV reactivation. [file 5097325.f1.docx]

Supplementary Table 1. Pretransplant cell populations according to the occurrence of mucositis, infection, and CMV reactivation.

| **Parameter** | **Mucositis (≥grade 3)** | | | **Infection before engraftment (CDI+MDI)** | | | **CMV reactivation** | | |
| --- | --- | --- | --- | --- | --- | --- | --- | --- | --- |
|  | **No**  **(n=43)** | **Yes**  **(n=16)** | **P** | **No**  **(n=30)** | **Yes**  **(n=29)** | **P** | **No**  **(n=42)** | **Yes**  **(n=17)** | **P** |
| MNC x10^6^ cells/ml, mean ± SEM | 1.26±0.11 | 0.96±0.12 | 0.128 | 1.28±0.15 | 1.11±0.11 | 0.364 | 1.18±0.11 | 1.16±0.13 | 0.917 |
| Frequency of cell population at engraftment (%), mean ± SEM | | | | | | | | | |
| CD3^+^ | 50.37±3.21 | 44.28±6.67 | 0.419 | 54.03±3.78 | 45.56±4.07 | 0.133 | 52.37±3.27 | 39.69±5.85 | 0.188 |
| CD16^+^CD56^+^ | 11.13±1.08 | 11.08±1.84 | 0.983 | 12.98±1.52 | 10.00±1.14 | 0.121 | 11.95±1.02 | 9.06±1.96 | 0.160 |
| HLA-DR^-^Lin^-^CD11b^+^CD33^+^ | 0.38±0.07 | 0.47±0.16 | 0.537 | 0.41±0.11 | 0.40±0.08 | 0.903 | 0.39±0.07 | 0.43±0.15 | 0.820 |
| HLA-DR^-^CD14^+^ | 2.19±0.54 | 2.91±0.92 | 0.494 | 2.41±0.67 | 2.38±0.63 | 0.975 | 2.55±0.60 | 1.99±0.66 | 0.593 |
| Absolute count of cell population, cells/μL, mean ± SEM | | | | | | | | | |
| CD3^+^ | 618.07±71.07 | 480.08±101.61 | 0.295 | 700.35±116.11 | 503.28±59.60 | 0.102 | 596.49±72.30 | 535.38±99.27 | 0.644 |
| CD16^+^CD56^+^ | 139.80±17.90 | 109.84±25.83 | 0.367 | 170.94±29.59 | 106.54±14.06 | 0.058 | 136.57±17.13 | 118.12±29.64 | 0.579 |
| HLA-DR^-^Lin^-^CD11b^+^CD33^+^ | 5.24±1.80 | 3.86±1.51 | 0.653 | 6.97±3.24 | 3.51±0.87 | 0.312 | 0.09±1.80 | 2.24±1.56 | 0.784 |
| HLA-DR^-^CD14^+^ | 22.11±6.03 | 29.01±10.95 | 0.562 | 30.49±10.45 | 19.99±5.58 | 0.338 | 27.49±7.23 | 15.22±2.55 | 0.116 |

MNC, mononuclear cell
